# Microbiome in Multiple Sclerosis: Where Are We, What We Know and Do Not Know

**DOI:** 10.3390/brainsci10040234

**Published:** 2020-04-14

**Authors:** Marina Kleopatra Boziki, Evangelia Kesidou, Paschalis Theotokis, Alexios-Fotios A. Mentis, Eleni Karafoulidou, Mikhail Melnikov, Anastasia Sviridova, Vladimir Rogovski, Alexey Boyko, Nikolaos Grigoriadis

**Affiliations:** 1 2nd Neurological University Department, Aristotle University of Thessaloniki, AHEPA General Hospital, 54634 Thessaloniki, Greece; bozikim@auth.gr (M.K.B.); bioevangelia@yahoo.gr (E.K.); ptheotokis@gmail.com (P.T.); elenikarafoulidou95@hotmail.com (E.K.); 2 Public Health Laboratories, Hellenic Pasteur Institute, Athens 11521, Greece; mentisaf@gmail.com; 3 Laboratory of Microbiology, University Hospital of Larissa, School of Medicine, University of Thessaly, 41110 Larissa, Greece; 4 Department of Neurology, Neurosurgery and Medical Genetics, Pirogov Russian National Research Medical University, Moscow 117997, Russia; medikms@yandex.ru (M.M.); anastasiya-ana@yandex.ru (A.S.); 5 Department of Neuroimmunology, Federal Center of Cerebrovascular Pathology and Stroke, Moscow 117342, Russia; 6 Department of Molecular Pharmacology and Radiobiology, Pirogov Russian National Research Medical University, Moscow 117997, Russia; qwer555@mail.ru

**Keywords:** gut microbiome, gut–brain axis, metagenomics, multiple sclerosis, disease-modifying treatments

## Abstract

An increase of multiple sclerosis (MS) incidence has been reported during the last decade, and this may be connected to environmental factors. This review article aims to encapsulate the current advances targeting the study of the gut–brain axis, which mediates the communication between the central nervous system and the gut microbiome. Clinical data arising from many research studies, which have assessed the effects of administered disease-modifying treatments in MS patients to the gut microbiome, are also recapitulated.

## 1. Introduction

The prevalence of multiple sclerosis (MS) has reportedly increased over the last few decades, showing both higher absolute numbers of patients and a real increase in MS incidence [1,2]. Overall, the number of patients afflicted with MS may be increasing due to a prolongation of their life expectancy and of the disease duration. Moreover, the integration of MAGNIMS (magnetic resonance imaging in multiple sclerosis) consensus in the diagnostic criteria for MS and the universal application of these criteria, together with their constant re-evaluation in order to achieve optimal sensitivity and specificity, allow for more accurate and early diagnoses [3,4]. The development of novel disease-modifying treatments (DMTs) that are effective in controlling disease activity and delaying progression (even in cases with highly active disease [5]), as well as the increase in physician’s awareness towards complications of the disease (such as spasticity, urinary disturbance, and chronic infections [6]), are also measures that increase life quality, and ultimately survival, for patients with MS. Moreover, the combined efforts of medical societies worldwide towards the development and application of universal registries and patient databases have led to improved case ascertainment that has also contributed to the observed increase of MS frequency [7]. In addition to the aforementioned advances in the health system and the medical services provided, a true increase in the incidence of MS has occurred in several ethnic populations over the last few decades. This is indicated by (i) the minimal number of ethnic populations that still remain free of the disease; (ii) a well-documented increase of the frequency of MS in previously low-incidence populations, such as in Asia, Southern and Eastern Europe; and (iii) the wider age window, i.e., younger than 16 and older than 50 years of age, in which the disease onset occurs [1,8,9]. According to the hygiene hypothesis, advanced civilization and technological progress in the recent past led to an improvement of the hygiene level of the overall life conditions for several ethnic populations and this improvement may be linked to increased MS frequency. In this respect, the observed alterations in MS incidence may be linked to an environmental shift towards a more MS-predisposing status. Another likely scenario is that the relative significance of the environmental factor with respect to MS pathogenesis has increased in the 21st Century. 

This review aims to summarize the recent advances that have been achieved in the analysis of gut microbiota, an environmental factor with a well-described impact in autoimmunity, and to provide a critical assessment of the derived knowledge with respect to the role of gut microbiota in MS pathogenesis. Moreover, we attempt to form key questions in order to position the derived knowledge into a valuable context with respect to personalized medicine and patient-tailored therapeutic approaches.

## 2. The Environmental Factor in Autoimmune Disease

A complex interplay between genetic and environmental factors is necessary for the development of autoimmunity. In MS, genetically predisposing factors have been recognized, with specific polymorphisms of the major histocompatibility complex (MHC), namely the human leukocyte antigen (HLA) system, to be the factors accounting for the majority of cases [10]. For instance, beta chain of HLA (HLA-DRB1 ) and DQ beta 1 chain of HLA (HLA-DQB1)polymorphisms have been implicated in MS predisposition in Caucasians. In addition, more than 130 single nucleotide polymorphisms (SNPs) implicated in various responses of the innate and adaptive immune system, as well as in cell survival and/or pathways of cellular death, have been recognized. However, even by considering the cumulative effect of these polymorphisms, the effect of the genetic factor itself does not account for more than 30% of MS cases [10]. Environmental factors have long been implicated in MS pathogenesis, and they include lifestyle conditions, such as smoking and the level of physical exercise, as well as the type of overall diet (e.g., Western, Eastern, or Mediterranean) and/or specific dietary parameters, such as vitamin D and salt intake [11,12]. Recently, it became evident that dietary and lifestyle conditions may exert a profound impact on the gastrointestinal tract (GI) and, more specifically, the intestine. This is an organ that appears to pose a significant role in regulating several responses of the signaling systems of the human organism, namely the endocrine, the immune, and, more remotely, the central nervous system (CNS) [13,14]. 

More importantly, the genetic factor itself is the main factor that is present upon the prenatal and immediate postnatal stage that determines predisposition towards disease at a level that remains relatively constant throughout life. Nonetheless, its outcome is subject to the effect of several environmental factors that are, overall, actively present throughout life. Each factor acts for an individual period of time and possibly affects a specific stage of disease pathogenesis, namely the predisposition, onset, and/or course of the disease [15]. According to the classical paradigm of genetics–environment interplay, gene polymorphisms are constant for a given individual and exert an effect upon their phenotype that remains constant throughout life. Environmental factors, on the other hand, continue to exert a biological effect that may be cumulative for the time period that the factor is present, or they may act as triggers that induce the onset of disease. An environmental factor may, therefore (i) act before the biological onset of the disease, thus contributing towards predisposition; (ii) act upon disease onset (trigger); or (iii) be present during the disease course, according to the LEARn (Latent Early Life-Associated Regulation) model, an epigenetic model of disease development described by Lahiri et al. Similarly, the GERSMS (Genetic and Environmental Risk Score) has been proposed as a means to quantify a combined estimate of an individual’s genetic burden and environmental exposures [16]. Special notice has been taken with respect to (i) the Western diet (ii) other lifestyle conditions, such as smoking, lack of physical excersise etc.; (iii) specific virus infections, such as the Epstein-Barr virus; (iv) the wide use of antibiotics; and (iv) the high sanitary level, as factors that promote pro-inflammatory responses; Several of these factors are present early (age <15 years old) in life [17].

## 3. Gut Microbiota and the Role of Intestinal Dysbiosis 

The human GI tract is colonized by approximately 1014 different populations of microorganisms. Overall, gut microbiota are nowadays regarded as a separate organ in the human body, weighing approximately 2 kg and carrying information that is at least 100 times larger than the number of human genes for an individual [18]. Under steady-state conditions, these microorganisms are symbiotic, in the sense that they contribute to the homeostasis of the human organism. More specifically, gut microbiota (i) contribute to the maintenance of the motility and permeability of the gut; (ii) prevent colonization by pathogens; (iii) mediate nutrient metabolism; (iv) participate in the production of vitamins, such as vitamin B complex, vitamin K, and folate; and (v) promote intestinal epithelial functions, such as absorption and secretion [18]. Recently, gut microbiota have been shown to shape the immune responses of innate and adaptive immunity, both locally (at the level of the GI mucosa) and systemically, thus affecting remote organs [19]. Data stemming from two large metagenomic databases, i.e., the MetaHIT (Metagenomics of the Human Intestinal Tract) and the Human Microbiome Project, isolated 2172 species in humans that were classified into 12 different phyla, with 93.5% of them belonging to the Proteobacteria, Firmicutes, Actinobacteria, and Bacteroidetes [20]. Large fractions of the phyla Firmicutes and Bacteroidetes reportedly include the genera *Prevotella*, *Bacteroides*, and *Ruminococcus*, and these are followed in size by Actinobacteria [20]. Moreover, the relative composition of the gut microbiota does not appear to be constant throughout different parts of the GI tract. Rather, there appears to be some degree of regional specialization with respect to the exact microbes that colonize each part of the gut [21]. For instance, frequent *Lactobacilli* are present in the duodenum, whereas both *Lactobacilli* and *Streptococci* are abundant in the jejunum. A large diversity has been described for the colon with the caecum and the appendix; these are two areas that also bear larger burden of microorganisms, in terms of absolute numbers [21]. Similarly, the diversity in microbiota across the GI tract leads to differential profiles of the metabolites that are produced as a result of the various microbiota mediating nutrient absorption and metabolism: In the stomach and the duodenum, vitamin A and aryl hydrocarbon receptors (AHR) ligands are primarily produced, whereas in the colon, a gradual shift towards higher short-chain fatty acid (SCFA) production is evident [22]. The structural architecture of the GI tract, as well as the differences in cellular composition and the pH of the adjacent mucosa, account for the alterations in the microbial composition and in the associated metabolites across the GI tract. Disequilibrium in the relative composition of intestinal microbiota has recently been recognized as a common underlying condition in several autoimmune diseases. The alteration of the intestinal microbial community that might lead to either animal or human diseases is termed intestinal or gut dysbiosis. Intestinal microbiota have been proven to shape immune responses and to affect the neural and endocrine systems of the gut. All these pathways exert remote signaling in the human body and thus bear implications for systemic and organ-specific autoimmunity, as in the case of the CNS [19]. 

## 4. The Gut Microbiota in MS

### 4.1. Immunoregulation and the Gut–Brain Axis

The enteric nervous system has long been recognized as a second brain. More recently, the gut–brain axis has been recognized as a bi-directional communication system from the CNS to the gut and vice versa; this communication is mediated by neuronal connections, neuroendocrine signals, general humoral signals, and immune signaling [23]. The CNS regulates gut function by promoting gut motility via a dense innervation system and by orchestrating local immune responses through the high numbers of immune cells that are present in the gut. These humoral signals are delivered by the utilization of common molecular mediators, such as pro-inflammatory cytokines, neuropeptides (like cholecystokinin (CCK) and leptin), and neurotransmitters (like dopamine (DA), serotonin (5-HT), gamma-aminobutyric acid (GABA), acetylcholine (Ach), and glutamate [22]). Conversely, structures in immediate proximity to the microbiota—such as the intestinal epithelial cells and immune cells in gut-associated lymphatic tissue (GALT) and the enteric nervous system (ENS)—mediate the transmission of signaling pathways from the gut towards the CNS. In this respect, gut microbiota may modulate the host via several pathways that originate in parts of the neuroendocrine, neural, and immune systems [23].

For instance, structurally distinct lipopolysaccharide (LPS), a characteristic component of the outer envelope of many microbes, exhibits a differential immunogenic profile in terms of the associated cytokines that are produced as a response by the host [24]. Toll-like receptor (TLR) signaling, a part of the pattern-recognition receptor (PRR) signaling, appears to be a key mediator of the host’s immune response towards bacteria, as it is the first-line sensing pathway that recognizes microbial structural patterns. 

Moreover, the recognition of bacterial structures by the TLR system prevents microbial translocation towards the deep layers of the gut lumen, as demonstrated in myeloid differentiation primary response 88 (MyD88) -/- mice that lack the expression of epithelial MyD88-dependent TLR [25]. In the bi-directional communication between the microbes and the host, it is therefore evident that the host may also regulate microbial colonization by the early recruitment of sensing and defense mechanisms. For example, cluster of differentiation antigen (CD) 1d (CD1d)+ invariant natural killer T (iNKT) cells and γδ intraepithelial lymphocytes (γδ IELs) are T-cell subsets that respond to microbial antigens. These cells were shown to regulate bacterial colonization in the gut [26]. Local immunoglobulin A (IgA) production by B-cells is also regarded as a factor regulating gut microflora composition and density [27,28]. Conversely, germinal center formation and the production of IgA are shaped by activation of T-follicular helper cells; the latter is induced by microbes and mediated by programmed cell death protein 1 (PD-1) [28].

### 4.2. Gut Microbiota and Innate Immunity 

Overall, microbiota are essential for priming the gastro-intestinal immune system to evoke specific immune responses: With respect to the innate immune system, several subsets of cells that participate antigen presentation respond to microbial stimuli by enhancing cytokine and chemokine production. The mucosa-associated invariant T (MAIT) cells, which express an invariant α T-cell receptor (TCR) chain and the non-classical MHC-I related protein located in mucosal tissues (e.g., intestinal lamina propria), produce diverse pro-inflammatory cytokines, such as interleukin (IL)-17, interferon gamma (IFNγ), granzyme B, or tumor necrosis factor alpha (TNFα) [29]. By expressing various chemokine receptors, MAIT cells exhibit a migratory capacity into remote tissues [29]. Natural killer (NK)-cells increase the expression of co-stimulatory molecules in response to microbial stimuli. NK cells are essential for the priming of other immune cells and the coordination of the overall host immune response by the production of IL-4, IL-13, and IFNγ, as well as the promotion of chemokine (C-X-C motif) ligand 16 (CXCL16) production by epithelial cells [29]. Dendritic cells and macrophages are, as is known, the classical antigen-presenting cells, and they play a key role in first-line host defense and the modulation of adaptive immunity. In so doing, they enhance the production of pro-IL-1β and its processing to bioactive IL-1β by caspase-1, thus discriminating between pathogenic and protective bacteria and dietary components [30].

### 4.3. Gut Microbiota and Adaptive Immunity 

The adaptive immune system also exhibits the capacity for microbe-driven responses. T-helper (Th)-17 cells are prevalent in the intestine, and they are important for the gastro-intestinal host defense, as they secrete cytokines that are involved in the regulation of inflammation (IL-17A, IL-17F, and IL-22). Specific microbes are capable of eliciting a differential T-effector phenotype (e.g., Th17 and Tregs) in the intestines and lymph nodes of mice that exhibit predisposition towards autoimmunity [31,32]. In germ-free mice or antibiotics-treated mice, the number of Th17 cells is reduced along with attenuated pro-inflammatory responses [33]. Moreover, mice that are resistant to autoimmunity exhibit the preferential sequestration of Th17 cells in the intestine, whereas peripheral blood Th17 repopulation by the administration of anti-(a4b7) integrin monoclonal antibodies rescues the autoimmune disease phenotype [34]. In this respect, the intestine appears to be a key regulating organ of immune system responses [34,35]. T regulatory (Treg) cells are two- to three-folds higher in abundance in the gastro-intestinal tract compared to other tissues. Mice with compromised gut microbiota, such as germ-free mice or antibiotic-treated mice, display a reduced frequency of Treg cells, and these Treg cells exhibit impaired anti-inflammatory cytokine-secretion, especially IL-10. In these mice, re-colonization by gut microbes promotes the function and frequency of Treg cells [32,36]. Moreover, bacterial antigens, such as LPS, are necessary for class-switch recombination in B cells towards IgA production. Additionally, B-cells primed by bacterial antigens have been shown to participate in antigen presentation and IgA selection in the germinal centers of the GALT (e.g., Peyer’s patches) [37].

### 4.4. The Role of Microbial Metabolites 

Apart from microbial structural components that may serve as antigens that shape immune responses in the intestine with implications for systemic disease, other molecular mediators also exhibit the capacity to induce pro- or anti-inflammatory reactions. Metabolites of microbial origin are present in the intestine, often as a by-product of nutrient degradation; these molecules may stimulate immune cells towards activated phenotype and cytokine production. SCFAs are metabolites produced by intestinal microbes that mediate a well-known anti-inflammatory effect. SCFAs inhibit histone deacetylases (HDACs) on Treg and microglia, a mechanism mediated by G-protein-coupled receptors (GPRs) [38]. Moreover, SCFAs may stimulate dendritic cells (DCs) towards the production of anti-inflammatory molecules, such as retinoic acid (RA) and transforming growth factor beta (TGFβ). Tryptophan metabolites evidently shape the phenotype of T cell subsets by promoting the production of either pro-inflammatory Th1 cytokines, such as IFN-γ and IL-2, or anti-inflammatory Th2 cytokines, such as IL-4 and IL-10 [38]. Tryptophan metabolites may also promote the Th17 pro-inflammatory phenotype by acting on AHR, a signaling pathway known to also affect astrocyte activation in the CNS as a response to microbial stimuli from the systemic circulation [38].

### 4.5. The Role of Intestinal Barrier 

Clinical and experimental evidence on the role of the intestinal barrier and its structural and functional integrity has recently elucidated aspects of the interplay between intestinal microbes and the host. An impaired intestinal barrier is regarded a common underlying condition in several autoimmune diseases of the gut that exhibit systemic implications, such as inflammatory bowel disease (IBD). In this respect, impaired intestinal barrier integrity exposes the cells of the local and, more importantly, of the systemic immune system to stimuli of microbial origin with a potential to elicit immune responses, as suggested in the context of the leaky gut theory. Intestinal dysbiosis appears to mediate barrier dysfunction, as this microbiome-related process may induce changes in mucus composition, enterocyte apoptosis and tight junction dysfunction through the translocation of associated structural components, as well as bacterial translocation to the lamina propria [39]. These alterations lead to an increased homing of lymphocytes in the lamina propria, the immunological layer of the intestinal barrier, and, in doing such, they contribute in the host’s predisposition towards local and systemic autoimmune responses. In the case of MS, such intestinal barrier alterations are linked to the presence of increased LPS and LPS-mediated signaling in the lamina propria, leading to chronic low-grade inflammation and endotoxemia [39]. Concomitant reduction in SCFAs associated to dysbiosis, that is, reduced microbial diversity, a condition frequently described in MS, results in compromized intestinal barrier and thus predisposes towards systemic pro-inflammatory reactions. In the CNS, microglia and astrocytes respond to pro-inflammatory stimuli from systemic circulation and acquire activated phenotypes, thus further promoting pro-inflammatory milieu in the context of CNS autoimmunity [39]. In this respect, the intestinal barrier has recently emerged as a novel target of pharmacological intervention in MS. This is because the restoration of the intestinal barrier may reduce the exposure of the cellular components of the systemic immune system to microbial derivatives and the associated pro-inflammatory cascade. 

### 4.6. Mechanisms of Immune-Modulation by Intestinal Microbiota—Experimental Evidence

Experimental evidence has dictated that the presence of intestinal microbiota is necessary in order for CNS autoimmunity to develop. In a myelin oligodendrocyte glycoprotein-specific t cell receptor (MOG-TCR) transgenic mouse model of spontaneous disease, experimental autoimmune encephalomyelitis (EAE) does not occur under germ-free (GF) conditions, whereas mice transferred from GF conditions into a conventional environment develop spontaneous EAE after few weeks of transfer [40]. Interestingly, MOG-TCR transgenic mice of a genetic background that is resistant towards autoimmunity, namely the B10.S mice, do not exhibit spontaneous EAE, even under conventional conditions [34]. In these mice, a preferential sequestration of Th17 pro-inflammatory T-cells in the intestine has been observed. The administration of anti-α4β7, a monoclonal antibody (mAb) that blocks intestinal integrin, has been found to be able to repopulate peripheral blood with Th17 T-cells and to rescue the disease phenotype [34]. The intestine thus appears as an organ with the ability to control systemic autoimmune responses with implication towards CNS autoimmunity [41]. In a similar context, the modulation of gut microbiota, as achieved by antibiotic administration, reduces the severity of conventional EAE. In an experimental setting, specific immune responses have been linked with single bacteria, such as in the case of Clostridia and *Bacteroides fragilis* derived from human feces that have the potential to induce Foxp3+ T regulatory cells, thus ameliorating EAE [42]. The fecal transplantation of MOG-TCR transgenic mice with human feces stemming from twins discordant for MS has only been found to result in the development of spontaneous EAE in mice recipients for feces stemming from twins with MS. In contrast, mice recipients for feces stemming from healthy twins have not been found to develop the disease [43]. GF mice recipients for the human feces of MS patients have also been found to develop severe EAE, coupled with alterations in the peripheral immune profile [44]. More specifically, fecal transplantation with material provided by healthy adults has been found to result in the induction of T regulatory cells in the mesenteric lymph nodes of the recipient mice, thus, overall, exerting an immune-regulatory response [44]. Conversely, the administration of *Lactobacilli* has been repeatedly shown to protect form EAE by the induction of IL-4, IL-10, TGF-β1, and IL-27 [45,46] by mediating an increase in IL-10+ and Foxp3+ T regulatory cells [47,48,49].

However, findings often observed in EAE fail to be translated to human disease due to the differences that the model exhibits. With the exception of spontaneous models, EAE requires myelin peptide immunization with a strong adjuvant. This is a condition that exerts especially skewed immune responses towards inflammation and results in a monophasic disease of inflammatory origin with little demyelination compared to the human disease [50]. In TCR transgenic mice that exhibit spontaneous disease, more than 90% of the circulating T-cells bear transgenic, autoreactive TCRs [51,52]. This is a condition that also does not accurately depict CNS autoimmunity in humans. In this respect, a detailed profiling of human microbiota appears to be a necessary approach to elucidate human-specific mechanisms. These mechanisms stem from interactions between the gut microbiota and the host, and they show the potential to induce autoimmune responses with relevance to the CNS.

### 4.7. Mechanisms of Immune-Modulation by Intestinal Microbiota—Clinical Evidence

During the last five years, several clinical studies have provided evidence indicating that in MS, the gut microbiome is altered. In an approach similar to the experimental model, initial studies linked alterations in the relative abundance of Clostridia, in the context of gut dysbiosis with MS. However, the clinical relevance—with respect to whether these alterations contribute towards susceptibility for MS or, instead, they exert a relative protective effect—remains controversial both for adult [53,54] and pediatric populations [55,56]. In 2016, two case control studies reported distinct patterns of gut microbiota composition by the use of 16S ribosomal ribonucleic acid rRNA metagenomics analysis [57,58] (for further discussion on the potential of metagenomic techniques as applied to MS, see [59,60]). These studies provided evidence of reduced diversity in the gut microbiome of MS patients compared to controls. Interestingly, this reduction was evident for patients with active MS, whereas patients in remission exhibited comparable diversity levels to the healthy population. Further studies verified this association of disease activity status with alterations of the relative abundance of microbes in the gut, with Firmicutes and Bacteroidetes exhibiting higher relative abundances reviewed by Kozhieva et al. [61]. These studies indicated the following: Though it is widely accepted that the gut microbiome in patients with MS is characterized by moderate dysbiosis, a clear and consistent multiple sclerosis microbiome phenotype has not been described. Moreover, given that a myriad of microbes have been implicated in MS, it is unlikely that, in the future, a single microbial organism will be isolated and characterized as an environmental trigger towards disease. This is in striking contrast with the paradigm stemming from mouse EAE. According to the latter, triggering of CNS autoimmunity by microbes provides mechanistic insight with respect to the molecular pathways that lead from the local immune responses in the gut to systemic inflammation and, eventually, to organ-specific autoimmunity towards the CNS [31,32]. 

Recently, a systematic review [62] of MS case-control studies with respect to gut microbiota composition concluded that, although differences in the diversity of microbiota were not reported by the majority of the included studies, several studies reported consistent patterns with respect to the taxonomic relative abundance. These findings further elucidated pattern alterations in the overall gut microbial composition of patients with MS compared to controls. Further prospective studies are necessary in order to establish a causative relation between these microbial pattern alterations and the disease pathogenesis and/or exacerbation. Notably, the majority of the reviewed studies referred to the Relapsing-remmitting type of MS(RRMS). A recent study addressed the differential gut microbiota profile in patients with primary progressive MS (PPMS), relatively to healthy controls [63]. As in the case of patients with RRMS, patients with PPMS have exhibited differences in a minority of a-diversity indices, whereas pattern differences have been observed at a taxonomic level [63].

In line with the observations described above, diet and dietary supplementation has recently emerged a major factor that affects gut microbiota’s relative composition. It has been proposed that a diet that is rich in vegetables, complex carbohydrates (fibers) combined with probiotics, vitamin D supplementation, vitamin A supplementation, and lipoic acid promotes gut eubiosis. This is coupled with a concomitant increase in microbial diversity and microbe-associated anti-inflammatory mediators, such as SCFAs, microbial anti-inflammatory molecules (MAMs), histone deacetylase inhibitors, AHR receptor agonists, and an increase of the Treg/Th17 ratio [64]. Conversely, a Western diet rich in animal fat and trans-fatty acids, with a high sugar and salt intake, promotes gut dysbiosis and results in (i) an increased presence of pro-inflammatory mediators such as TNFa, IL-6, and IL-17; and (ii) increased gut barrier and blood–brain barrier (BBB) permeability with implications for systemic and CNS autoimmunity [64]. More specifically, gut dysbiosis predisposes one to intestinal inflammation, which is characterized by alterations in the immunological barrier layer of the lamina propria towards pro-inflammatory milieu in the GALT and an increase in the presence of endotoxin/LPS in the intestinal mucosa. The further translocation of LPS and other bacterial components, as well as whole bacteria in the deep layers of the intestinal wall and the local secondary lymphoid organs (such as the mesenteric lymph nodes) allows for the generation of circulating activated T-cells in the context of low-grade endotoxemia [39,64]. These systemic alterations (i) compromise the integrity of the BBB, (ii) allow for pro-inflammatory stimuli to cross the BBB towards the CNS, and (iii) affect microglia and astrocyte activation status, thus predisposing one towards neuroinflammation.

Clinical evidence of the possible causal relationship between the gut microbiota profile and the CNS autoimmunity stems from more interventional approaches that actively alter gut microflora composition; such approaches include fecal microbiota transplantation (FMT), an investigational method that has been used successfully to treat cases of severe enterocolitis [65,66] More specifically, prolonged antibiotic administration in certain individuals may cause expansion of *Clostridium difficile (C. difficile)* at the expense of symbiotic bacteria, thus serving as an example of intestinal dysbiosis and *C. difficile*-related severe enterocolitis. FMT protocols require that fecal material from a healthy donor, following careful donor screening and appropriate preparation procedures, is transferred to a patient, either via colonoscopy or via an oral route as capsule ingestion [67]. Due to risks linked with transplantation (i.e., possible transmittable disease) and colonoscopy procedures, FMT is reserved for cases that are refractory to the antibiotics that are typically prescribed against enterocolitis due to *C. difficile*. Recently, FMT has been advocated as an attractive therapeutic approach for several diseases that are linked to intestinal dysbiosis, either of the intestine, such as IBD [68,69] or systemic extragastric and CNS disease [70,71] (and reviewed in [72]). Isolated case reports have described the beneficial effects of FMT over MS disease course, and a clinical trial of FMT for patients with MS is currently underway [73]. 

## 5. Disease-Modifying Treatment (DMT) and Gut Microbiota

In the management of MS, DMTs serve as prophylactic treatments towards clinical and radiological disease activity, whereas other medications are prescribed for symptom management. The later are more often prescribed in the context of disability accumulation, such as gamma-Aminobutyric acid-type B GABA_B_ receptor agonists (e.g., baclofen) for limb spasticity and a-adrenergic inhibitors for the control of overactive bladders. Several of these regimens are known to alter the profile of the gut microbiota [39]. Recently, several oral DMTs were shown to inhibit the growth of *Clostridium* in vitro. This feature has been proposed to contribute to the DMTs’ overall anti-inflammatory mechanism of action [74,75]. In this study, fingolimod was proven to be bactericidal, whereas teriflunomide and dimethyl fumarate (DMF) exerted a bacteriostatic effect. Clinical data stemming from metagenomics analysis of gut microbiota alterations in patients receiving DMF and glatiramer acetate (GA) further verified that DMTs exert a profound effect on the relative composition of gut microbiota. The above could shed light into potential additional mechanisms of action [76].

### 5.1. Interferon-β

With respect to IFNβ, several lines of investigation have indicated that it may modify the immunological properties of the intestinal barrier. IFNβ is a member of the type 1 interferon (T1IFN) family, and it is considered a major cytokine that mediates local responses to viral, bacterial, and other antigen stimuli in the intestine (reviewed in [77]). In a mouse model of pneumococcal lung infection, IFNβ treatment led to the upregulation of tight junction proteins in lung epithelial and blood vessel endothelial layers, thus reducing lung–blood barrier permeability and preventing invasive pneumococcal infection [78]. Furthermore, IFNβ, produced by DCs following stimulation by gut commensal microbiota, was recently shown to mediate Treg proliferation in the intestine [79]. A clinical case-control study exploring the effect of IFNβ administration in patients with MS recently reported an increase of *Prevotella*, a known probiotic, in patients with MS treated with IFNβ. This increase was comparable to healthy controls, whereas untreated patients exhibited a reduced relative abundance of probiotics [80].

### 5.2. Glatiramer Acetate

GA is a myelin-basic protein (MBP) analog and a long-administered first-line DMT treatment for MS. In line with its anti-inflammatory properties, GA is known to ameliorate colonic injury in an experimental model of colitis by inducing a reduction in TNFa and a concomitant increase in Tregs, IL-10, and TGFb-producing cells [81]. Moreover, the role of GA in stabilizing the intestinal barrier and promoting tissue repair, as documented by analysis of syndecan-1 expression, was shown in a model of IBD [82]. In EAE, GA administration ameliorated the disease phenotype coupled with an increase in gut *Prevotella*, and the administration of GA combined with GI colonization with live *Prevotella* led to the further attenuation of disease [83]. GA treatment in patients with MS was shown to exert an effect in the relative abundance of gut microbiota, especially the Lachnospiraceae and Veillonellaceae families [76]. Similarly, another case-control study in patients with RRMS treated with GA reported alterations in the relative composition of the gut microbiome with respect to several *Clostridium* [84].

### 5.3. Dimethyl Fumarate

DMF is a long-prescribed DMT for psoriasis that was more recently approved as a first-line treatment for MS. In an experimental setting, DMF was shown to promote an increase in the relative abundance of probiotics and to stabilize the intestinal barrier. A concomitant increase in SCFA-producing microbes was shown to further promote the systemic anti-inflammatory effect of the drug [85]. Similarly, DMF administration in Lewis rats was shown to mediate (i) a reduction in the TLR-4 expression by the GALT, (ii) a reduction in IFNγ, and (iii) a concomitant increase in lamina propria’s Foxp-3+ expression and the abundance of CD4+CD25+ Tregs in Peyer’s patches [86]. A case-control study implementing metagenomics techniques reported an association between DMF treatment and decreased relative abundance of Firmicutes and Clostridia, as well as an increase of Bacteroidetes, relative to untreated patients [76].

### 5.4. Teriflunomide

Teriflunomide, another oral, first-line DMT approved as a prophylactic treatment for RRMS, has been shown to modify immune responses in the intestine by promoting the local proliferation of CD39+ Tregs. Thus, it exerted anti-inflammatory action that ameliorated CNS inflammation in a mouse model of EAE [87]. 

### 5.5. Natalizumab

Natalizumab (NTZ) is an injectable second-line DMT that has been approved for patients with highly active RRMS. NTZ is a monoclonal antibody targeting a4-integrin, a family that includes adhesion molecules expressed in T-cells. Integrins exhibit tissue specificity with a4b1 expressed in the CNS, whereas a4b7 is expressed in the intestine. NTZ is not selective; therefore, by inhibiting T-cell trafficking towards the CNS, it also blocks T-cell circulation in the gut. In addition to its beneficial effect in MS, NTZ has been shown to be effective in ameliorating the symptoms of IBD [88]. The administration of NTZ has been proposed as preferred treatment approach for patients with RRMS and IBD co-morbidity [88]. Furthermore, IBD is a well-described condition characterized by intestinal dysbiosis and local immune dysregulation. In this respect, also in patients with RRMS that do not exhibit signs of IBD, the amelioration of T-cell trafficking in the gut by NTZ may contribute to the drug’s anti-inflammatory effect by inhibiting the circulation of activated T-cells in the gut. The gut has recently been proposed as a regulating organ with respect to the peripheral circulation of activated T-cells, with implications for CNS autoimmunity. In mice resistant to EAE that exhibit the preferential sequestration of autoreactive Th17 T-cells in the intestine, the administration of the a4b7 mAb led to the re-population of peripheral blood with Th17 T-cells and rescued the disease phenotype [34]. Interestingly, in this MOG-TCR transgenic mouse model, the selective accumulation of autoreactive T-cells in the intestine acts as a mechanism of immune tolerance that contributes in resistance towards EAE [34]. In the context of MS, it is reasonable to assume that the blocking of T-cell trafficking in the intestine may ameliorate the exposure of the immune system to stimuli of microbial origin. This amelioration could be performed by reducing antigen sampling and, subsequently, T-cell clonal expansion and activation in response to these antigens. As gut dysbiosis, and the associated local immune dysregulation, is frequently reported in patients with RRMS, this concomitant effect may serve as an additional mode of action for NTZ in RRMS.

### 5.6. Fingolimod

Fingolimod is an oral second-line DMT that is indicated as prophylactic treatment for highly active RRMS. Fingolimod is a sphingosine-1-phosphate (S1P) agonist, acting on four out of five S1P receptors, on various organs and cell types. Fingolimod ligation on S1P receptors leads to the downregulation of the S1P receptor expression; therefore, the drug is considered as a functional antagonist of S1P signaling. On a physiological level, S1P receptor expression is necessary for the lymphocytes, either naïve or activated, to egress from the secondary lymphoid organs, such as the peripheral lymph nodes. Due to this mechanism of action, fingolimod inhibits the egress of lymphocytes from lymph nodes to the peripheral blood stream. Thus, it ameliorates systemic immune responses and CNS-targeted autoimmunity. Similarly, other S1P ligands have been tested for intestinal autoimmune disease, such as IBD, as they exhibit the potential to ameliorate the transmigration of immune cells across the intestine [89]. Fingolimod has been shown to ameliorate experimental colitis [90], and two S1P ligands are currently being tested in terms of safety and efficacy in phase II and phase III clinical trials on colitis [88]. Interestingly, S1P signaling has been shown to regulate innate lymphoid cell (ILC) transmigration from intestinal lamina propria towards systemic circulation and other lymphoid organs, thus regulating infectious and inflammatory responses [91]. In a transgenic mouse model of enteric nervous system pathology resembling Parkinson’s disease (PD) due to a-synuclein accumulation, fingolimod resulted in an enhanced gut motility and increased levels of brain-derived neurotrophic factor (BDNF) [92]. Moreover, S1P ligation has been shown to exert a stabilizing effect towards barrier function [93,94] and the BBB [95], with implications for EAE and MS [96,97,98]. Fingolimod may potentially exert an effect on the gut microbiome’s relative composition, as it has been shown to regulate IgA plasmablasts’ maturation from the intestinal Peyer’s patches, a first-line defense mechanism of the host towards microbe colonization [99,100]. Moreover, fingolimod was shown to exert a direct anti-microbial effect by inhibiting the growth of *Clostridium* and the associated endotoxin production in vitro [74].

### 5.7. Alemtuzumab

Alemtuzumab is an anti-CD52 monoclonal antibody inducing T-cell and B-cell depletion in peripheral blood. It was originally used for the treatment of chronic B-cell lymphocytic leukemia. Recently, alemtuzumab has been approved for the treatment of highly active RRMS for patients with breakthrough disease that were previously exposed to other first- and/or second-line DMTs [101]. Apart from the obvious effect of alemtuzumab in depleting circulating primarily B- and T-cell lymphocytes, the long-term immunomodulating effect is mediated by alterations that the drug causes in the peripheral immune cell pool following repopulation. Some of these alterations are attributed to the homeostatic proliferation of mature lymphocytes that the drug promotes in peripheral tissues [102]. Due to this effect, the administration of alemtuzumab has been linked with an increased susceptibility towards autoimmune comorbidities, such as autoimmune thyroid disease, membranous glomerulonephritis, autoimmune hepatitis, and immune thrombocytopenic purpura [103]. Colitis due to *Clostridium* was the cause of fatal outcome in one patient with RRMS who received alemtuzumab [104], whereas another patient presented with pancolitis during the first course of alemtuzumab treatment [105]. Susceptibility towards infection was the assumed underlying cause in both cases. With respect to the second case, an immune-mediated mechanism contributing to sepsis has also been proposed [105]. Evidence stemming from a cynomolgus monkey model indicated that the intestinal barrier may be disrupted during alemtuzumab treatment [106]. In macaques monkeys, a single dose of alemtuzumab resulted in (i) intestinal epithelial cell loss, (ii) increased apoptosis in the villi, and (iii) an abnormal Paneth cell morphology [107]. Mouse anti-CD52 mAb also resulted in increased numbers of IELs undergoing apoptosis and disrupted intestinal barrier function in mice [108]. In a cynomolgus monkey model, alemtuzumab administration resulted in profound alterations in the relative composition of gut microbiota, namely Lactobacillales, Enterobacterales, and Clostridiales, as well as the genera *Prevotella* and *Faecalibacterium*. These alterations were primarily linked to alterations in the relative abundance of TCRαβ+ or TCRγδ+ T cells [109]. These data indicate that alemtuzumab administration exerts a profound effect on the intestinal homeostasis with respect to tissue integrity, barrier function, immune properties, and microbiome profile. However, it remains unknown whether these alterations are beneficial in the context of CNS autoimmunity, or, instead, if, in a proportion of patients, the overall beneficiary effect of alemtuzumab in subsiding disease activity is counterbalanced by a detrimental effect in intestinal function. 

## 6. Conclusions: Treat the Microbiome—Treat MS?

The combined efforts of the scientific community in the field of MS have been focused on identifying strategies that may be implemented in order to either modify the peripheral immune responses or, as proposed by the less successful approach to date, to enhance neuroprotection and the endogenous regenerative capacity of the CNS. In addition to the classical paradigm of immune–brain interaction in the context of MS, the intestine has emerged as an additional regulating organ of responses that take place both in the immunological and the nervous (central and peripheral) counterparts. In this respect, the gut commensal microbiota may serve as environmental factors that shape the intestinal milieu. The modification of gut microbiota by either dietary (e.g., probiotic supplementation) or medicinal approaches (e.g., antibiotic administration) may serve as additional therapeutic strategies for MS prophylaxis [110]. More interventional approaches, such as FMT, have also been proposed. Moreover, the relative composition of gut microbiota may also serve as an indicator of reciprocal host–microorganism interactions. Further longitudinal studies that implement the profiling of intestinal microbiota during the pre-clinical phase and over the course of the disease are needed in order to elucidate this assumption. MS is a complex autoimmune disease with clinical variability. As such, the establishment of a causative role for intestinal microbiota towards disease pathogenesis requires combined efforts from the field of metagenomics and other “-omics” approaches [59,60,111] with the capacity for high throughput data production and the application of these data in the context of translational medicine.

With respect to future directions, we consider gut microbiota modulation as a promising intervention for the management of MS. Understanding the pathways that the gut microbiota implicate in order to shape host’s immune responses may elucidate therapeutic targets, such as the induction of immune regulatory cell populations via the promotion of an “anti-inflammatory” gut microflora. Similar interventions, possibly in combination with DMTs, may contribute in promoting treatment efficacy and optimal response. As several newly available DMTs confer significant and potentially severe adverse effects, gut microbiota modification has emerged as a promising, and possibly less interventional, additional approach. 
